# Tunable Quantum Tunneling through a Graphene/Bi_2_Se_3_ Heterointerface for the Hybrid Photodetection Mechanism

**DOI:** 10.1021/acsami.1c18606

**Published:** 2021-12-02

**Authors:** Hoon Hahn Yoon, Faisal Ahmed, Yunyun Dai, Henry A. Fernandez, Xiaoqi Cui, Xueyin Bai, Diao Li, Mingde Du, Harri Lipsanen, Zhipei Sun

**Affiliations:** † Department of Electronics and Nanoengineering, Aalto University, FI-00076 Aalto, Finland; ‡ QTF Centre of Excellence, Department of Applied Physics, Aalto University, FI-00076 Aalto, Finland

**Keywords:** tunable quantum tunneling, graphene, topological insulator, heterointerface, asymmetric barrier, hybrid photodetection

## Abstract

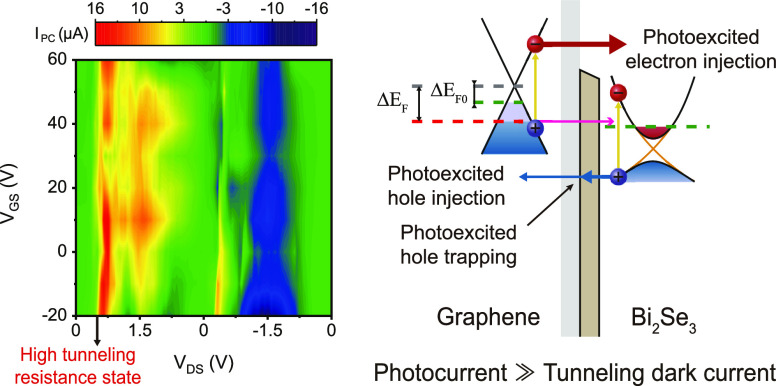

Graphene-based van der Waals heterostructures are promising building blocks for broadband photodetection because of the gapless nature of graphene. However, their performance is mostly limited by the inevitable trade-off between low dark current and photocurrent generation. Here, we demonstrate a hybrid photodetection mode based on the photogating effect coupled with the photovoltaic effect via tunable quantum tunneling through the unique graphene/Bi_2_Se_3_ heterointerface. The tunneling junction formed between the semimetallic graphene and the topologically insulating Bi_2_Se_3_ exhibits asymmetric rectifying and hysteretic current–voltage characteristics, which significantly suppresses the dark current and enhances the photocurrent. The photocurrent-to-dark current ratio increases by about a factor of 10 with the electrical tuning of tunneling resistance for efficient light detection covering the major photonic spectral band from the visible to the mid-infrared ranges. Our findings provide a novel concept of using tunable quantum tunneling for highly sensitive broadband photodetection in mixed-dimensional van der Waals heterostructures.

## Introduction

Light detection over a wide spectral range, from the visible to the mid-infrared, has a great potential for numerous photonic and optoelectronic applications. In this scenario, graphene can provide a versatile platform for broadband photodetection due to its gapless electronic band structure.^1−4^ However, its low absorption and fast recombination of photogenerated carriers result in low photocurrent with comparably large dark current.^5−7^ These limitations present major challenges, restraining the practical applications of photodetectors based on graphene. Various methods have been introduced to overcome limitations by enhancing the light absorption of graphene-based photodetectors, including plasmonic nanostructures^8^ and quantum dots.^9^ Although these approaches have unique advantages, their photodetection is limited to a short spectral range because of the sharp resonant absorption.^10,11^

Hybrid graphene systems combined with mixed-dimensional materials for high-sensitivity broadband light detection have emerged from recent developments by assembling materials into van der Waals heterostructures.^12,13^ In graphene-based van der Waals heterostructures, semiconducting two-dimensional transition-metal dichalcogenide materials are typically used as they exhibit superior light–matter interaction properties that allow for enhanced light absorption. However, the absorption is limited within the visible spectral range due to their band gap.^14^ Combining graphene with narrower band gap materials has been proposed for broader light absorption despite the large dark current.^15−17^ Among them, most of the studies using topological insulators focused on the formation of graphene/topological insulator heterostructures to induce the photogating effect, utilizing the novel properties of topological insulators such as graphene-like hexagonal symmetry, direct narrow band gap, and ultrafast photocurrent along the surface.^18,19^ As a material candidate to form van der Waals heterostructures with graphene, topological insulators have advantages beyond the aforementioned properties. For example, compared to black phosphorus, which is similar to a topological insulator in terms of band gap and electrical properties such as mobility and carrier multiplication, the surface oxidation of black phosphorus is related to the degradation mechanism and environmental instability,^20^ while the surface oxidation of the topological insulators serves to protect the surface states.^21,22^ Moreover, since the topological insulators are Dirac fermion materials like graphene, a Dirac-source field-effect transistor can be realized with the graphene/topological insulator heterostructures.^23^

Recent studies reported the control over the dark current in graphene-based photodetectors by introducing an interlayer (e.g., h-BN) tunneling barrier with enhanced photodetectivity.^5−7^ However, the photodetection performance using this method is highly sensitive to the size, thickness, and quality of the interlayer, which makes the fabrication process challenging. As an alternative pathway free from introducing an interlayer, the natural oxidation layer rapidly formed on the surface of topological insulators^21,22^ in the ambient environment can be utilized as the tunneling barrier.^24−26^ In particular, the thickness of the oxidation layer of the topological insulators is saturated within a few hours after exfoliation, and the oxidation process is significantly delayed over time so that a uniform oxidation layer can be obtained over the entire surface.^21,22^

Here, we demonstrate that the naturally formed oxidation layer at the graphene/Bi_2_Se_3_ heterointerface enables incorporation of the quantum tunneling effect into the photodetection mechanism, as evidenced by the transition of charge carrier transport mechanisms from direct tunneling to Fowler–Nordheim (FN) tunneling and/or thermionic emission. In our device architecture, the photogating effect in the graphene/Bi_2_Se_3_ heterochannel is coupled to the photovoltaic effect through the rectifying tunneling junction. This significantly enhances the photodetection performance, which is fundamentally different from the typical graphene-based photodetectors previously reported.^1−4^ Accordingly, the normalized photocurrent-to-dark current ratio (NPDR) is enhanced by around an order of magnitude via electrical tuning of tunneling resistance for detection of light covering the major photonic spectral region from the visible to the mid-infrared wavelengths. Our work provides a new perspective on both the tunneling dark current suppression and the efficient photocurrent generation for various photonic and optoelectronic applications.

## Results and Discussion

### Graphene/Bi_2_Se_3_ Heterojunction Device

**Figure 1:**
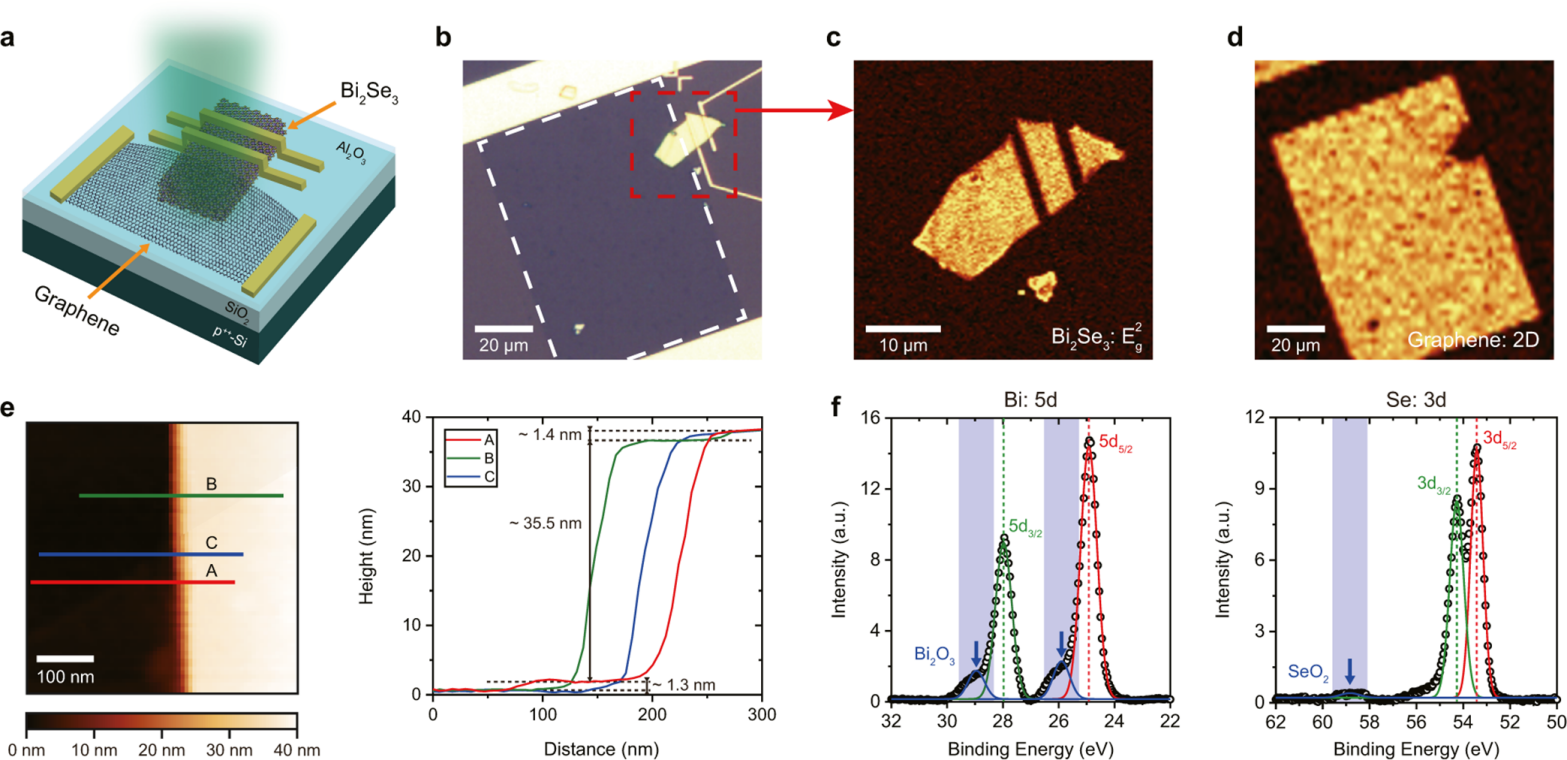
Graphene/Bi_2_Se_3_ heterojunction device. (a) Schematic illustration of the device structure. (b) Optical microscope image of the graphene/Bi_2_Se_3_ heterochannel. The red dashed square represents the heterojunction region. The white dashed square outlines the graphene channel. (c,d) Raman mapping images for the *E*_g_^2^ peak intensity of Bi_2_Se_3_ (c) and the 2D peak intensity of graphene (d). (e) Atomic force microscopy (AFM) image (left) and its line scan profiles (right). The black arrows (right panel) indicate the thicknesses of the Bi_2_Se_3_ flake and graphene layer. (f) X-ray photoemission spectroscopy (XPS) spectra measured on Bi_2_Se_3_ with Bi 5d (left) and Se 3d (right) peaks. The oxidation peaks (Bi_2_O_3_ and SeO_2_) are indicated by the blue arrows.

Our Dirac-source field-effect transistor based on a lateral heterochannel and a vertical tunneling junction is realized by the graphene/Bi_2_Se_3_ heterostructure (see the methods/experimental section for the fabrication details). As shown in Figure 1a, our devices feature a long striped graphene channel in contact with a Bi_2_Se_3_ flake on the side. Graphene acts not only as a passivation layer to protect the tunneling junction but also as an efficient charge carrier transport channel. The insulating bulk states of the bottom Bi_2_Se_3_ flake combined with the top Al_2_O_3_ insulating layer enable us to investigate the mechanism of carrier transport through the interfacial barrier between the conducting Dirac surface states of graphene and Bi_2_Se_3_. The device is characterized by optical microscopy (Figure 1b) and Raman spectroscopy (Figures 1c,d and S1). The *E*_g_^2^ peak intensity mapping image of the Bi_2_Se_3_ flake (marked with the red dashed square in Figure 1b) is shown in Figure 1c. The Raman signal of the Bi_2_Se_3_ flake on the graphene/Bi_2_Se_3_ heterojunction region is almost similar to that on the region without the graphene layer (see Figure S1a). On the other hand, the 2D peak intensity mapping image of graphene, as shown in Figure 1d, is not revealed on the heterojunction region since the Raman signal of graphene is significantly reduced on the heterojunction region compared to that on the region without the Bi_2_Se_3_ flake (see Figure S1b).

The graphene layer covering the surface around the edge of Bi_2_Se_3_ flake can be clearly identified by the AFM images, as shown in Figures 1e and S2. The average thicknesses of the graphene and Bi_2_Se_3_ flakes, measured by AFM, are ∼1.3 and 29.2 nm, respectively (see the Supporting Information for more details on the AFM)^27−29^.

Figure 1f shows the XPS on Bi_2_Se_3_ (Bi: 5d and Se: 3d) taken after 24 h from exfoliation. The observed oxidation peaks corresponding to Bi_2_O_3_ (at around 26 and 29 eV) and SeO_2_ (at around 59 eV) represent the existence of the oxidation layer naturally formed on the Bi_2_Se_3_ surface.^22^ The intensity of the SeO_2_ peak is much lower than that of the Bi_2_O_3_ peak, indicating that the dominant oxidation layer formed on the Bi_2_Se_3_ surface is Bi_2_O_3_ rather than SeO_2_ due to the Se vacancies on the Bi_2_Se_3_ surface.^30−32^ Note that the oxidation time of 24 h after exfoliation under ambient conditions is set to utilize the uniform oxidation layer with the stabilized thickness. Although the formation of the native oxidation layer is very fast at the initial stage after exfoliation,^21,22^ its thickness is known to saturate since the oxidation process is significantly delayed over time due to the interplay between surface exposure and oxygen incorporation.^22^ The thickness of the oxidation layer is estimated as ∼2 nm (±0.2 nm).^21,22,24−26^

### Tunable Quantum Tunneling through the Heterointerface

The van der Waals heterostructures can enable versatile functionalities with higher performance than each material in the van der Waals heterostructures.^12,13^ First, we investigated the current–voltage (*I*–*V*) characteristics of the graphene/Bi_2_Se_3_ heterojunction by choosing different metal electrodes (see the methods/experimental section for the measurement details). When the source and drain are applied across the graphene/Bi_2_Se_3_ heterointerface (defined as graphene-Bi_2_Se_3_), the *I*–*V* curves exhibit the nonlinear *I*–*V* relationship (Figure S3c) due to charge carrier transport through the graphene/Bi_2_Se_3_ heterointerface. The asymmetric rectifying behavior indicates that the tunneling junction is formed at the interface, and the tunneling barrier heights are asymmetric. The hysteresis effect of *I*–*V* curves arises from charge trapping at the interface. For comparison, we also measure a reference graphene transistor (defined as graphene&Bi_2_Se_3_), where both source and drain are applied to the graphene channel that partially covers the Bi_2_Se_3_ flake (see the Supporting Information for details on the measurement configuration with different electrodes). The *I*–*V* curves, as shown in Figure S3a, reveal the typical Ohmic behavior in the reference graphene transistor.

**Figure 2:**
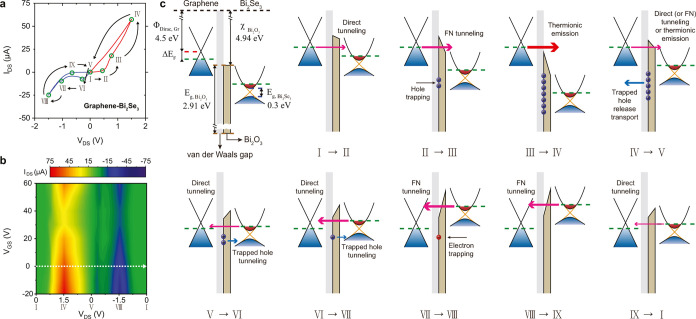
Tunneling through the heterointerface and transition of carrier transport mechanisms. (a) *I*_DS_–*V*_DS_ curve of the graphene-Bi_2_Se_3_ at *V*_GS_ = 0 V, which consists of nine different operation regimes from I to IX. (b) Color plots of *I*_DS_ depending on *V*_DS_ and *V*_GS_. The white dotted arrow in (b) represents the path of steps in the *I*_DS_–*V*_DS_ curve (a). (c) Energy band alignments of the graphene/Bi_2_Se_3_ interface before equilibrium and between each step (Φ_Dirac,Gr_: work function of intrinsic graphene when the Fermi level is at Dirac point, Δ*E*_F_: Fermi-level shift from the Dirac point of graphene, *E*_g,Bi_2_O_3__: Bi_2_O_3_ band gap, χ_Bi_2_O_3__: Bi_2_O_3_ electron affinity, and *E*_g,Bi_2_Se_3__: Bi_2_Se_3_ band gap). The gray and brown areas represent the van der Waals gap and Bi_2_O_3_ layer, respectively. The color of arrows represents the carrier transport mechanisms (magenta: direct or FN tunneling, red: thermionic emission, and blue: trapped hole release or tunneling), and the thickness of arrows indicates the relative amount of current. The red and blue circles are electron and hole carriers, respectively. The red, green, and blue dashed lines represent the Dirac point of graphene, the Fermi-level of graphene or Bi_2_Se_3_, and the conduction or valence band edge of Bi_2_Se_3_, respectively.

**Figure 3:**
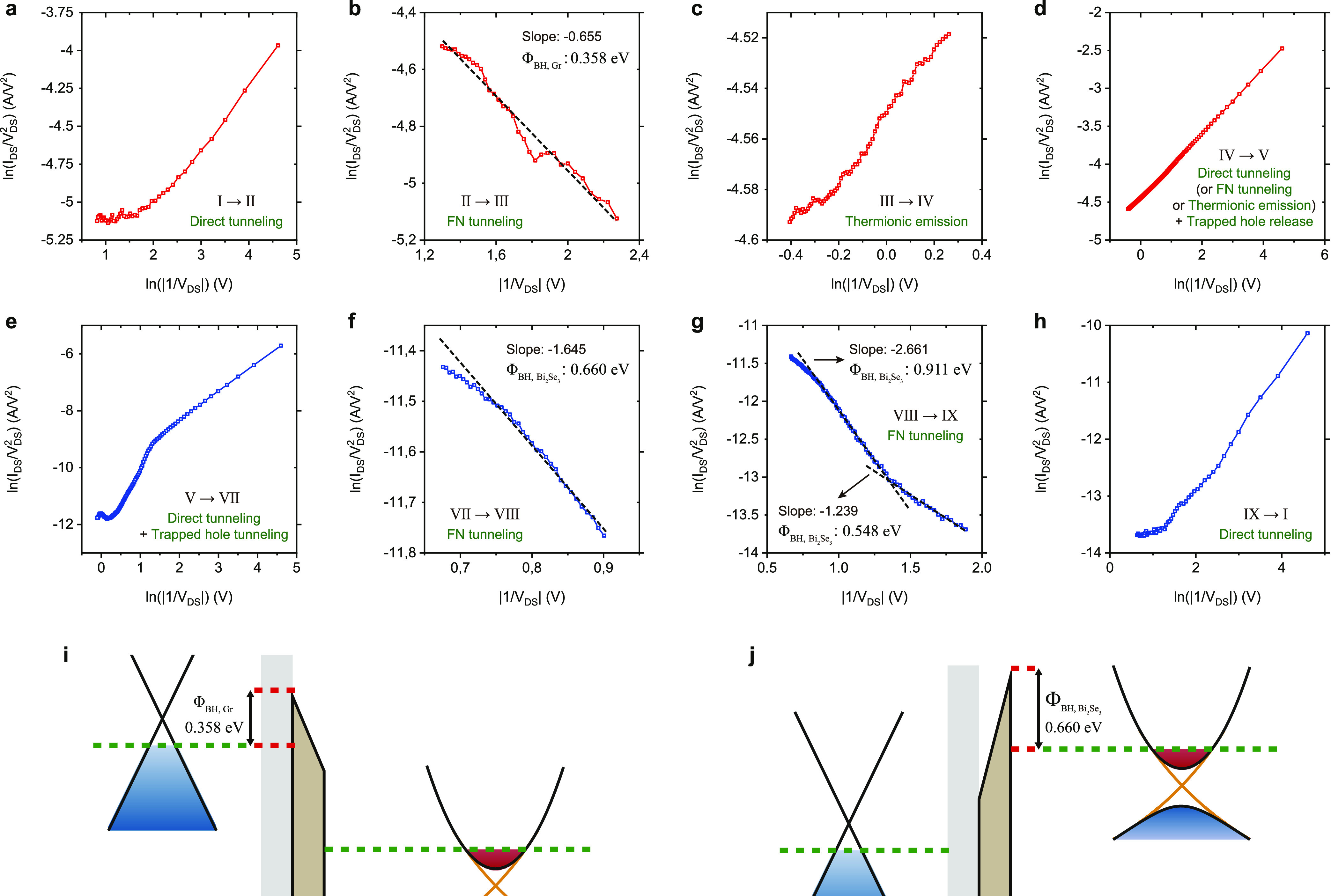
Asymmetric tunneling barrier heights. (a–h) FN plots of the graphene-Bi_2_Se_3_ for *V*_DS_ > 0 (a–d) and *V*_DS_ < 0 (e–h). (i,j) Band alignments across the graphene/Bi_2_Se_3_ interface when the FN tunneling occurs in *V*_DS_ > 0 (i) and *V*_DS_ < 0 (j). Each barrier height is extracted from (b,f) respectively.

To understand the mechanism of asymmetric rectifying and hysteretic characteristics of the graphene-Bi_2_Se_3_, as shown in Figure 2a,b, the *I*_DS_–*V*_DS_ curve (drain–source current *I*_DS_ as a function of drain–source voltage *V*_DS_) at gate–source voltage *V*_GS_ = 0 V is divided into nine steps, which are marked by Roman numerals from I to IX. Each step represents the transition point, where the transport mechanism changes and the resistance state switches to different resistance states. The oxidation layer naturally formed on the Bi_2_Se_3_ surface is known to act as a tunneling barrier in contact with graphene.^21,22,24−26^ The tunneling resistance is closely related to the potential barrier at the interface and the electronic density of states in graphene and Bi_2_Se_3_. Hence, the shape deformation of the asymmetric tunneling barrier will have a great influence on the tunneling current across the interface. The energy band alignments before equilibrium and between each step is drawn in Figure 2c based on the estimation of the dominant transport mechanism, as shown in Figure 3, by fitting Figure 2a to the direct or FN tunneling equations^33−36^ (see the Supporting Information for details on the FN tunneling plot analysis). The detailed descriptions for the hysteretic *I*–*V* characteristics and charge carrier trapping processes at the graphene/Bi_2_Se_3_ interface can also be found in the Supporting Information.^37,38^ Further details on the energy band alignment are fully discussed in the Supporting Information.^30−32,39−46^

### Coupling the Photogating Effect with the Photovoltaic Effect

Most graphene-based photodetectors generally focus on one photodetection mechanism: photovoltaic effect, photogating effect, photo-thermoelectric effect, and bolometric effect, due to their inherent limitations.^1−4^ On the contrary, in our device, the photogating effect is coupled to the photovoltaic effect through the rectifying tunneling junction across the graphene/Bi_2_Se_3_ heterointerface, which is supported by the observation of asymmetric rectifying and hysteretic *I*–*V* characteristics, as shown in Figure 2a,b. The photogating effect is known to stem from the change in channel resistance and carrier density due to photogenerated carriers, which can be induced by charge trapping at or charge transfer across the interface.^47−49^ Some of the photogenerated carriers accumulated at the trap states can act as an external bias voltage to shift the Fermi-level of graphene, and the other carriers injected into graphene or Bi_2_Se_3_ will contribute to the photocurrent. On the other hand, the photovoltaic effect is driven by separating photogenerated electron–hole pairs through the rectifying junction, which can be further controlled by tuning the tunneling resistance.^5−7^ Before exploring the photogating effect, we first characterized the enhanced photocurrent with the photovoltaic effect, owing to the rectifying tunneling junction, as shown in Figure S3. Interestingly, under the same condition of light illumination (at a wavelength of 532 nm with a laser power of 10 μW) focused onto the same graphene/Bi_2_Se_3_ heterojunction region, much higher photocurrent is realized through the graphene/Bi_2_Se_3_ heterointerface (Figure S3f, graphene-Bi_2_Se_3_), as compared to the reference graphene transistor (Figure S3e, graphene&Bi_2_Se_3_). This is because the photocurrent generated in the reference graphene transistor is hindered by the carrier recombination within the graphene channel, while the photocurrent of the rectifying tunneling junction formed through the graphene/Bi_2_Se_3_ heterointerface is enhanced with the photovoltaic effect.

**Figure 4:**
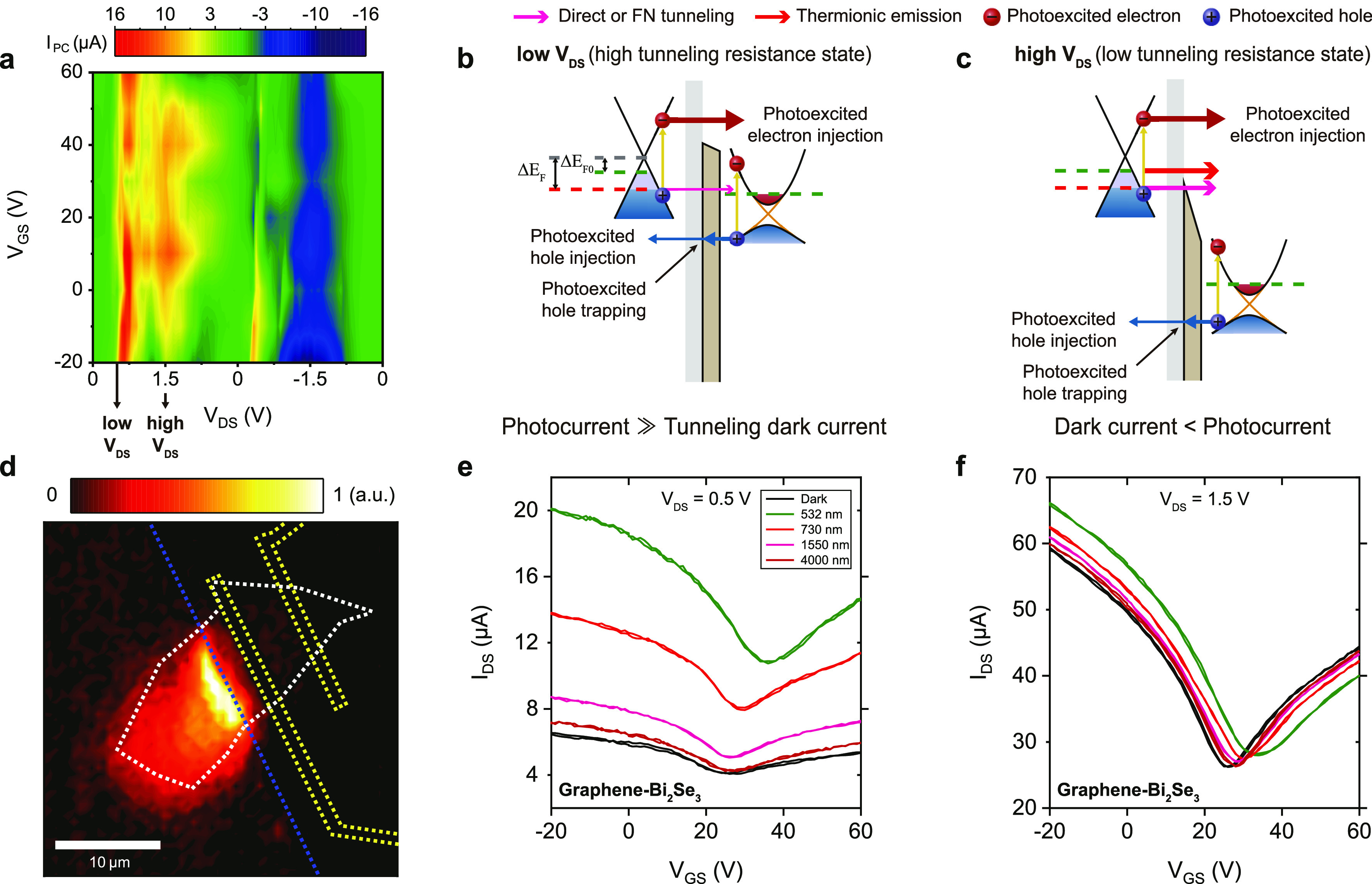
Tunable photoresponse across the graphene/Bi_2_Se_3_ heterointerface. (a) Color plots of *I*_PC_ depending on *V*_DS_ and *V*_GS_. (b,c) Operation principle of the photodetection mechanism. Δ*E*_F0_ and Δ*E*_F_ are the Fermi-level shift from the Dirac point of graphene in the dark and under light illumination, respectively. (d) Photocurrent mapping plot measured near the heterojunction at *V*_DS_ = 0.5 V applied across the graphene/Bi_2_Se_3_ heterointerface and *V*_GS_ = 0 V under 532 nm light illumination (100 μW). The blue, white, and yellow dotted lines outline the graphene layer, Bi_2_Se_3_ flake, and Ti/Au electrodes, respectively. (e,f) *I*_DS_–*V*_GS_ curves at *V*_DS_ = 0.5 (e) and *V*_DS_ = 1.5 (f) in the dark or under light illumination over a wide range of wavelengths (532, 730, 1550, and 4000 nm) with a power of 10 μW. The black arrow in (a) represents the selected *V*_DS_ (0.5 and 1.5 V) for descriptions in (b,c) and *I*_DS_–*V*_GS_ curves in (e,f).

We also find that the tunneling resistance can be significantly tuned by varying *V*_DS_ in our graphene/Bi_2_Se_3_ heterochannel due to the strong coupling between the photogating and photovoltaic effects. Here, two different *V*_DS_ (0.5 and 1.5 V) are chosen to define the high and low tunneling resistance states. Both exhibit high photocurrents, but dark currents are obtained to be considerably different for a proper comparison. Note that this is based on the color plots of *I*_PC_, as shown in Figure 4a, where *I*_PC_ = *I*_light_ – *I*_dark_ is the photocurrent, *I*_light_ is the drain–source current under light illumination, and *I*_dark_ is the drain–source current in the dark. In addition, it is found to be more effective for modulating the tunneling resistance by tuning positive *V*_DS_ along the Bi_2_Se_3_ side, due to the lower barrier height for electrons on the graphene side than that toward the Bi_2_Se_3_ side, as estimated in Figure 3. The operation principle is described in Figure 4b,c, incorporating the tunneling process into the photodetection mechanism. At the high tunneling resistance state (Figure 4b, *V*_DS_ = 0.5 V), the direct tunneling from graphene to Bi_2_Se_3_ will be substantially blocked by the tunneling barrier under the dark condition, while the photoexcited electrons in graphene can be easily injected into Bi_2_Se_3_ over the low barrier height. On the other hand, at the low tunneling resistance state (Figure 4c, *V*_DS_ = 1.5 V), the dark current ascribed to the FN tunneling and thermionic emission will exceed the current due to photoexcited electrons. As a result, the dark current will be obtained to be extremely lower in the high tunneling resistance state (Figure 4b) than that in the low tunneling resistance state (Figure 4c). As shown in Figure 4d, the scanning photocurrent measurements are carried out to investigate the spatial photoresponse in the graphene/Bi_2_Se_3_ heterojunction at *V*_DS_ = 0.5 V (at a wavelength of 532 nm with a laser power of 100 μW). The photocurrent generation is pronounced around the heterojunction region especially near the edge of the graphene channel overlapping the Bi_2_Se_3_ flake, confirming the major photocurrent generation originating from the heterointerface due to the built-in electric fields applied across it.

### Optical Switching Ratio Enhancement

The transfer curves (*I*_DS_ as a function of *V*_GS_) of the graphene/Bi_2_Se_3_ heterointerface in the dark and under light illumination at various wavelengths of 532, 730, 1550, and 4000 nm with a light power of 10 μW are shown in Figure 4e (*V*_DS_ = 0.5 V) and Figure 4f (*V*_DS_ = 1.5 V). The graphene/Bi_2_Se_3_ heterochannel operates as a field-effect transistor, where the carrier mobility depends on the tunneling resistance. The high (*V*_DS_ = 0.5 V) and low (*V*_DS_ = 1.5 V) tunneling resistance states lead to the different average carrier mobilities of 36.8 and 103.2 cm^2^ V^–1^ s^–1^ for holes and 12.6 and 64.1 cm^2^ V^–1^ s^–1^ for electrons at room temperature, respectively. From the shift of *V*_Dirac_, we estimated that the photogating effect is attributed to the trapping of photogenerated carriers at the graphene/Bi_2_Se_3_ interface (see the Supporting Information for details on the photogating effect due to the trapping of photogenerated carriers). As shown in the transfer curves, there are almost no hysteresis effects for the *V*_GS_ sweep, thanks to the Al_2_O_3_ top passivation layer,^50^ implying that charge trapping at the graphene/Bi_2_Se_3_ interface only occurs during the *V*_DS_ sweep. In particular, the tunneling dark current is obtained to be quite low, as shown in Figure 4e, giving rise to noticeable enhancement of the optical switching ratio (*I*_light_/*I*_dark_). At *V*_GS_ = 0 V, although the photocurrents (*I*_PC_ = *I*_light_ – *I*_dark_), as shown in Figure 4e,f, are similar to each other, the dark current is measured to be much larger in Figure 4f than that in Figure 4e. This is consistent with the interpretation provided in Figure 4b,c. The zero-crossing point of *I*_PC_, where *I*_light_ = *I*_dark_, does not appear in Figure 4e, indicating that the photovoltaic effect contributes to the ratio of *I*_light_ to *I*_dark_. Unlike the photogating effect in which one type of photogenerated carriers should be captured in trap states, the photovoltaic effect requires the efficient separation of created electron–hole pairs. Our results suggest that the tunneling junction in the graphene/Bi_2_Se_3_ heterochannel can be utilized to couple the photogating effect with the photovoltaic effect to enhance the optical switching ratio by tuning the tunneling resistance.

**Figure 5:**
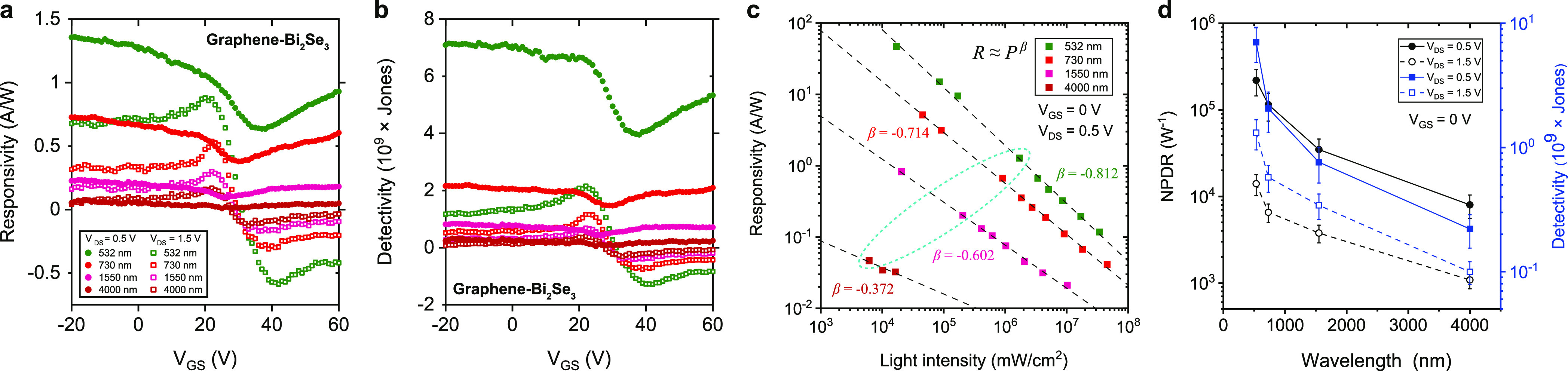
Photoresponse characteristics depending on tunneling resistance. (a,b) Photoresponsivity (a) and photodetectivity (b) plots of the graphene-Bi_2_Se_3_ as a function of *V*_GS_ at *V*_DS_ = 0.5 and 1.5 V over a wide range of wavelengths (532, 730, 1550, and 4000 nm) with an incident light power of 10 μW. (c) Responsivity dependence on incident light intensity. The black dashed lines are the linear fitting to the data. (d) NPDR and photodetectivity as a function of wavelength at *V*_DS_ = 0.5 and 1.5 V. The turquoise dashed circle in (c) represents the group of light intensities used in (d).

Depending on the tunneling resistance, the electrically tunable photoresponse of graphene-Bi_2_Se_3_ is confirmed by photoresponsivity (Figure 5a, *R* = *I*_PC_/*P*_effective_) and photodetectivity (Figure 5b, ), where *P*_effective_ is the effective light power illuminated onto the actual photoactive area *A*_active_ after considering the input beam waist, and the total area of graphene channel and Bi_2_Se_3_ flake to avoid the overestimation of photodetectivity. The photodetectivity of the graphene/Bi_2_Se_3_ heterointerface (Figure 5b) can be effectively maximized by tuning *V*_DS_ to 0.5 V (the high tunneling resistance state). On the contrary, the photoresponse characteristics of the reference graphene transistor (Figure S4c,d) is almost unchanged between *V*_DS_ = 0.5 and 1.5 V. In other words, the light absorption in the graphene/Bi_2_Se_3_ heterostructure does not guarantee the high photodetectivity. This highlights that the charge carrier transport through the graphene/Bi_2_Se_3_ tunneling junction is of great importance to couple the photogating effect with the photovoltaic effect in the graphene/Bi_2_Se_3_ heterochannel for highly sensitive photodetection.

The dependence of photoresponsivity on light intensity (*I*) at *V*_DS_ = 0.5 V and *V*_GS_ = 0 V is plotted in Figure 5c (see the Supporting Information for details on the relation between light power and responsivity).^51^Figure 5d shows that the tunneling resistance at which the values of NPDR and photodetectivity become maximum can be electrically tuned. The NPDR are enhanced from ∼1.4 × 10^4^, 6.6 × 10^3^, 3.8 × 10^3^, and 1.1 × 10^3^ W^–1^ (at *V*_DS_ = 1.5 V) to ∼2.2 × 10^5^, 1.1 × 10^5^, 3.5 × 10^4^, and 8.0 × 10^3^ W^–1^ (at *V*_DS_ = 0.5 V) by effectively suppressing the tunneling dark current. The idea of tuning the tunneling resistance for enhancing the photodetectivity offers a new insight to realize the broadband photodetection by coupling the photogating effect with the photovoltaic effect.^52^

In other studies, several attempts have been made to control the dark current by utilizing the ultrathin interlayer such as Ta_2_O_5_^5^ and h-BN^6,7^ as the tunneling barrier. However, their tunneling resistance has been usually controlled by the interlayer thickness, which was set during the fabrication process. In our work, the unique band alignment across the graphene/Bi_2_Se_3_ interface^30−32,43−46^ leads to the transition from direct tunneling at the low bias voltage (high tunneling resistance state) to FN tunneling and/or thermionic emission at the high bias voltage (low tunneling resistance state), as described in Figure 4b,c. This allows us to modulate the tunneling dark current just by varying the bias voltage across the graphene/Bi_2_Se_3_ junction. As an alternative pathway free from an artificial introduction of an interlayer at the interface, our findings demonstrate a new perspective of utilizing the oxidation layer naturally formed at the graphene/Bi_2_Se_3_ interface for both dark current reduction and efficient photocurrent generation. We confirmed that the naturally formed oxidation layer can act as a tunneling barrier. We further observed the tunable tunneling resistance and offered direct evidence for the asymmetric tunneling barrier heights using the tunneling equations.^33−36^ An additional advantage of utilizing the naturally formed oxidation layer is that this approach is not limited by the size, thickness, and quality of the interlayer so that large-scale devices are achievable as long as Bi_2_Se_3_ is large enough, making the entire fabrication process simple. This is similar to the current silicon technology, where naturally formed silica plays a key role. Another novelty of our work is describing the transition of charge carrier transport mechanisms through the graphene/Bi_2_Se_3_ interface, which is essential for coupling between photogating and photovoltaic effects. To the best of our knowledge, the hysteresis effect of *I*–*V* curves in graphene/Bi_2_Se_3_ heterojunction is first observed in this work, implying the trapping of charge carriers at the interface. This observation suggests that the trap-assisted photogating effect can be induced in the graphene/Bi_2_Se_3_ heterochannel by trapping of photogenerated carriers,^47−49^ which can be coupled with the photovoltaic effect to effectively enhance the photocurrent owing to the rectifying tunneling junction.^5−7^

## Conclusions

Based on the asymmetric tunneling barrier formed at the graphene/Bi_2_Se_3_ interface,^24−26^ we have explored a breakthrough way to improve the photoresponse characteristics of the graphene/Bi_2_Se_3_ heterochannel by suppressing the tunneling dark current and injecting the photogenerated carriers. We have found that the tunneling resistance of the graphene/Bi_2_Se_3_ heterojunction can be tuned to couple the photogating effect with the photovoltaic effect in the graphene/Bi_2_Se_3_ heterochannel. The transition of charge carrier transport mechanisms through the graphene/Bi_2_Se_3_ interface is a key signature to modulate the optical switching ratio.^5−7^ The practical application to improve the device performance through the interface engineering (e.g., asymmetric tunneling barrier height, interface-trap density, and Dirac surface states) or the material combination (e.g., other topological insulators or other materials of small band gap), which is beyond the scope of this study, will be a promising research direction. This study will provide useful information for designing novel photodetectors for highly sensitive broadband photodetection.

## Methods/Experimental Section

### Sample Preparation and Device Fabrication

Our heterojunction phototransistor was fabricated by integrating graphene and Bi_2_Se_3_ into van der Waals heterostructures. The Bi_2_Se_3_ flakes were mechanically exfoliated from bulk material and transferred onto the highly doped Si substrates with a 285 nm thick SiO_2_ layer preprocessed with solvent cleaning and O_2_ plasma treatment. The Bi_2_Se_3_ flake surface was naturally oxidized under ambient conditions for 24 h. Owing to the rapid surface oxidation of topological insulators,^17,21,22^ there was no need to introduce an additional interlayer such as an insulating layer grown by atomic layer deposition (ALD) or h-BN layer before the graphene transfer. In order to form several heterostructures on each substrate at the same time, large-area monolayer graphene grown by chemical vapor deposition, purchased from Graphenea, was wet-transferred onto the substrates.^39−42^ For the complete and smooth coverage of the graphene layer over the entire Bi_2_Se_3_ flake, we selected the Bi_2_Se_3_ flakes with a few tens of nanometer thick, which allowed us to minimize the defects caused by the steep surface morphology around the flake edges. Each graphene channel was patterned to a regular shape with electron beam lithography (EBL, Vistec EBPG 5000) and reactive ion etching (Oxford Instruments PlasmaLab 80 Plus). The lateral heterochannel was defined to be the patterned graphene ribbon and transferred Bi_2_Se_3_ flake, while the vertical tunneling junction was formed in the overlapping regions. The heterojunction area was estimated to range from 50 to 100 μm^2^, with an average of 83.7 μm^2^. Ti/Au electrodes of 5/75 nm were patterned with EBL and deposited through electron beam evaporation (MASA IM-9912) followed by a lift-off process. The metal electrodes on the graphene channel and Bi_2_Se_3_ flakes were patterned to be orthogonal and parallel to the graphene channel, respectively. As a passivation layer, a 10 nm thick layer of Al_2_O_3_ grown by ALD (ALD, Beneq TFS-500) at 150 °C was used to prevent the surface modification from the adhesion of oxygen or water molecules. After completing the device fabrication, an optical microscope (Olympus BX60) and Micro-Raman (WITec Alpha 300 RA+) system using 532 nm continuous wave laser were used to characterize the graphene/Bi_2_Se_3_ heterostructure. The graphene covering the Bi_2_Se_3_ flake edge was identified by AFM Dimension Icon (Bruker). XPS was performed on the large area Bi_2_Se_3_ flakes using a Kratos Axis Ultra ESCA spectrometer with a monoenergetic Al Kα (1486.96 eV) source. The pass energy was ∼20 eV, and the X-ray spot size was ∼200 μm. Since X-rays penetrate only the top few layers of flakes, the XPS is useful for quantitative analysis of the surface chemical composition (the outer few nanometers) regardless of the flake thickness.

### Experimental Details and Electro-Optical Measurement Setup

The sample holder was designed in a size fitting into a fixing holder in a probe stage. After device fabrication, the device chips were mounted onto each printed circuit board (PCB) attached to the sample holder and wire-bonded to the PCB. All the electrical measurements were carried out in an optical microscope (WITec Alpha 300 RA+) or a home-built femtosecond laser based microscopic system with two sourcemeters (Keithley 2400 and 2401) at room temperature under ambient conditions. The gate voltage was applied to the Si substrate, while the source and drain voltages were applied to the metal electrodes connected to the graphene channel or Bi_2_Se_3_ flakes. The photocurrent measurements covering from the visible range to near-infrared range were conducted in the WITec system combined with two sourcemeters. The light beams from continuous wave lasers at 532 nm (WITec focus innovations), 730 nm (Thorlabs MCLS1), and 1550 nm (Photonetics) were focused onto the heterojunction through an objective lens (100×, NA = 0.75). The optical microscopy platform system allowed us to focus the laser beam on desired positions in the sample. The diameters of the light spot were around 0.87, 1.18, and 2.49 μm, respectively. The photocurrent measurements in the mid-infrared range were conducted in a home-built femtosecond laser-based microscopic system combined with two sourcemeters. The duration and repetition rate of the incident pulse were 230 fs and 2 kHz. The laser at 4000 nm is focused to cover the graphene/Bi_2_Se_3_ heterojunction region by a parabolic mirror. The diameter of the light spot was ∼20 μm.

## Supporting Information Available

Raman spectra measured near the heterojunction region; AFM characterization of the graphene layer and Bi_2_Se_3_ flake; graphene-Bi_2_Se_3_ and graphene&Bi_2_Se_3_; FN tunneling plot analysis; charge carrier trapping processes at the interface; energy band alignment across the graphene/Bi_2_Se_3_ heterointerface; photoresponse characteristics of the graphene&Bi_2_Se_3_; output and transfer curves of the Bi_2_Se_3_ field-effect transistor; photogating effect due to the trapping of photogenerated carriers; relation between light power and responsivity; and photoswitch characteristics of the graphene-Bi_2_Se_3_ (PDF)
