# Supplementary material for: Tunable Quantum Tunneling through a Graphene/Bi2Se3 Heterointerface for the Hybrid Photodetection Mechanism
Source: ACS Appl Mater Interfaces. 2021 Dec 2;13(49):58927–35. doi: 10.1021/acsami.1c18606 (PMC8678989; doi:10.1021/acsami.1c18606)
Supplement: Supplementary file 1 — am1c18606_si_001.pdf [file am1c18606_si_001.pdf]

Supporting Information: Tunable Quantum Tunneling through a Graphene/Bi_2Se_3 Heterointerface for the Hybrid Photodetection Mechanism

Hoon Hahn Yoon,[*,†,‡] Faisal Ahmed,[†] Yunyun Dai,[†,‡] Henry A. Fernandez,[†,‡] Xiaoqi Cui,[†,‡] Xueyin Bai,[†,‡] Diao Li,[†,‡] Mingde Du,[†,‡] Harri Lipsanen,[†] and Zhipei Sun[*,†,‡]

†*Department of Electronics and Nanoengineering, Aalto University, FI-00076 Aalto, Finland*

‡*QTF Centre of Excellence, Department of Applied Physics, Aalto University, FI-00076 Aalto, Finland*

E-mail: hoonhahn.yoon@aalto.fi; zhipei.sun@aalto.fi

Raman spectra measured near the heterojunction region

The Raman signal of the Bi_2Se_3 flake measured on the graphene/Bi_2Se_3 heterojunction region (green in **Figure S1**a) is almost similar to that on the region without top graphene layer (blue in **Figure S1**a). On the other hand, the 2D peak intensity mapping image of graphene in **Figure 1**d is not revealed on the heterojunction region, since the Raman signal of graphene is significantly reduced on the heterojunction region (green in **Figure S1**b) compared to that on the region without Bi_2Se_3 flake (red in **Figure S1**b).

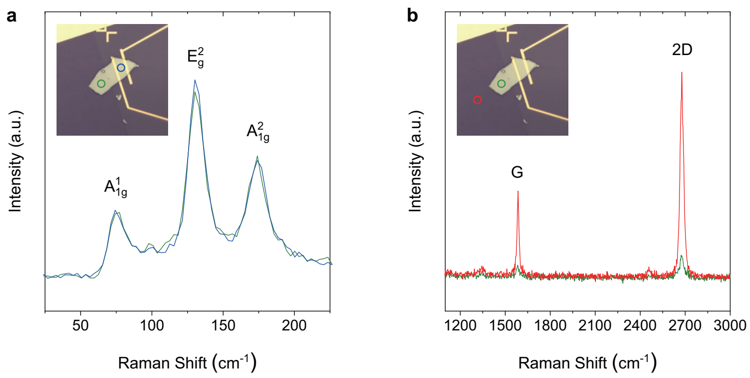

Figure S1: Raman spectra measured near the heterojunction region. **a**, Raman spectra obtained from the Bi_2Se_3 flake on the graphene/Bi_2Se_3 heterojunction region (green) and the region without top graphene layer (blue). **b**, Raman spectra obtained from the graphene layer on the heterojunction region (green) and the region without Bi_2Se_3 flake (red). Raman spectroscopy was carried out under illumination of a 532 nm continuous wave laser. Each inset shows the optical image near the heterojunction and marks the region where each Raman spectra was taken.

AFM characterization of the graphene layer and Bi_2Se_3 flake

We investigated the surface morphology with atomic force microscopy (AFM) to check the graphene layer covering the surface around the edge of Bi_2Se_3 flake. **Figure S2**a shows the AFM image (top) near the Bi_2Se_3 flake edge with its zoom-in views (middle: 2D and bottom: 3D) on the region indicated by the magenta square in the top image. The graphene layer located below the boundary (indicated by the yellow line) can be clearly identified in the zoom-in views. The thickness can be measured from the line scan profiles in **Figure S2**b. The average thicknesses of the graphene layer and Bi_2Se_3 flakes, measured by AFM, are 1.3 nm and 29.2 nm, respectively. Note that the Bi_2Se_3 flakes with thicknesses of several tens of nanometers are found suitable for investigating the photoresponse properties in the graphene/Bi_2Se_3 heterojunction, while maintaining both the conductive surface states and bulk bandgap nature of Bi_2Se_3. This is likely because the insulating states are dominated in ultrathin Bi_2Se_3 films below approximately 3 quintuple layers (QL), and 4-12 QL thick flakes still suffer from low conductivity limited by edge transport.[1–3] However, for the very thick Bi_2Se_3 flakes ($>$ 100 nm), we found that it is very difficult to completely and smoothly cover the Bi_2Se_3 flakes with graphene layer due to the defects caused by steep surface morphology around the flake edges.

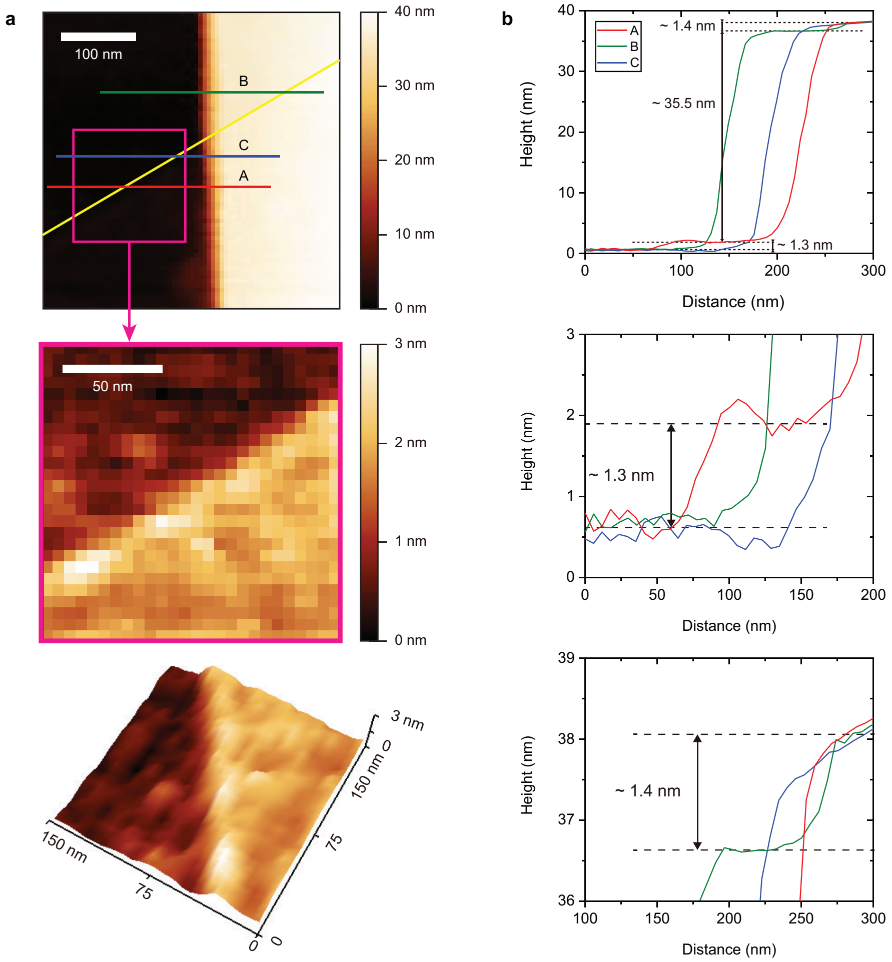

Figure S2: AFM characterization of graphene layer around Bi_2Se_3 flake edge. **a**, AFM mapping image (top) and its zoom-in views (middle: 2D and bottom: 3D). The yellow line represents the edge of graphene layer. **b**, Line scan profiles of A-C indicated in **a** (A: red, B: green, C: blue). The black arrows indicate the thicknesses of Bi_2Se_3 flake and graphene layer.

Graphene-Bi_2Se_3 and graphene&Bi_2Se_3

As shown in **Figure S3**, the current-voltage (I-V) characteristics (drain-source current I_{DS} as a function of drain-source voltage V_{DS} depending on gate-source voltage V_{GS}) of the graphene/Bi_2Se_3 heterojunction were investigated by choosing different metal electrodes in dark or under light illumination on the heterojunction region (at a wavelength of 532 nm with a laser power of 10 μW) onto the graphene/Bi_2Se_3 heterojunction. One is a reference graphene transistor (graphene&Bi_2Se_3), where both source and drain are applied to the graphene channel that partially covers the Bi_2Se_3 flake (**Figure S3**a,b,e). Another is the graphene-Bi_2Se_3 heterochannel (graphene-Bi_2Se_3), where source and drain are applied across the graphene/Bi_2Se_3 heterointerface (**Figure S3**c,d,f). The I-V curves of the reference graphene transistor reveals the typical Ohmic behavior, while that of the graphene-Bi_2Se_3 heterochannel exhibits the asymmetric rectifying and hysteretic characteristics. Each color plot of I_{DS} depending on V_{DS} and V_{GS} is shown below. For the graphene&Bi_2Se_3, although the side Bi_2Se_3 flake will affect the local resistance change of graphene due to charge transfer at the interface, most of the current is observed to flow through the graphene channel (**Figure S3**a), and it is difficult to recognize the change in current under 532 nm laser illumination (**Figure S3**b). On the one hand, the nonlinear I-V relationship of the graphene-Bi_2Se_3 is attributed to charge carrier transport through the graphene/Bi_2Se_3 heterointerface. The asymmetric rectifying I-V curves (**Figure S3**c) indicate that the tunneling junction is formed at the interface, and the barrier heights are asymmetric. The hysteresis effect of I-V curves arises from charge trapping at the interface. Owing to the rectifying behaviors, the current increase under 532 nm laser illumination is recognizable in the graphene-Bi_2Se_3 heterochannel. The color plots of photocurrent are provided in **Figure S3**e,f. The photocurrent is measured much higher in the graphene-Bi_2Se_3 heterochannel (**Figure S3**f) than the reference graphene transistor (**Figure S3**e).

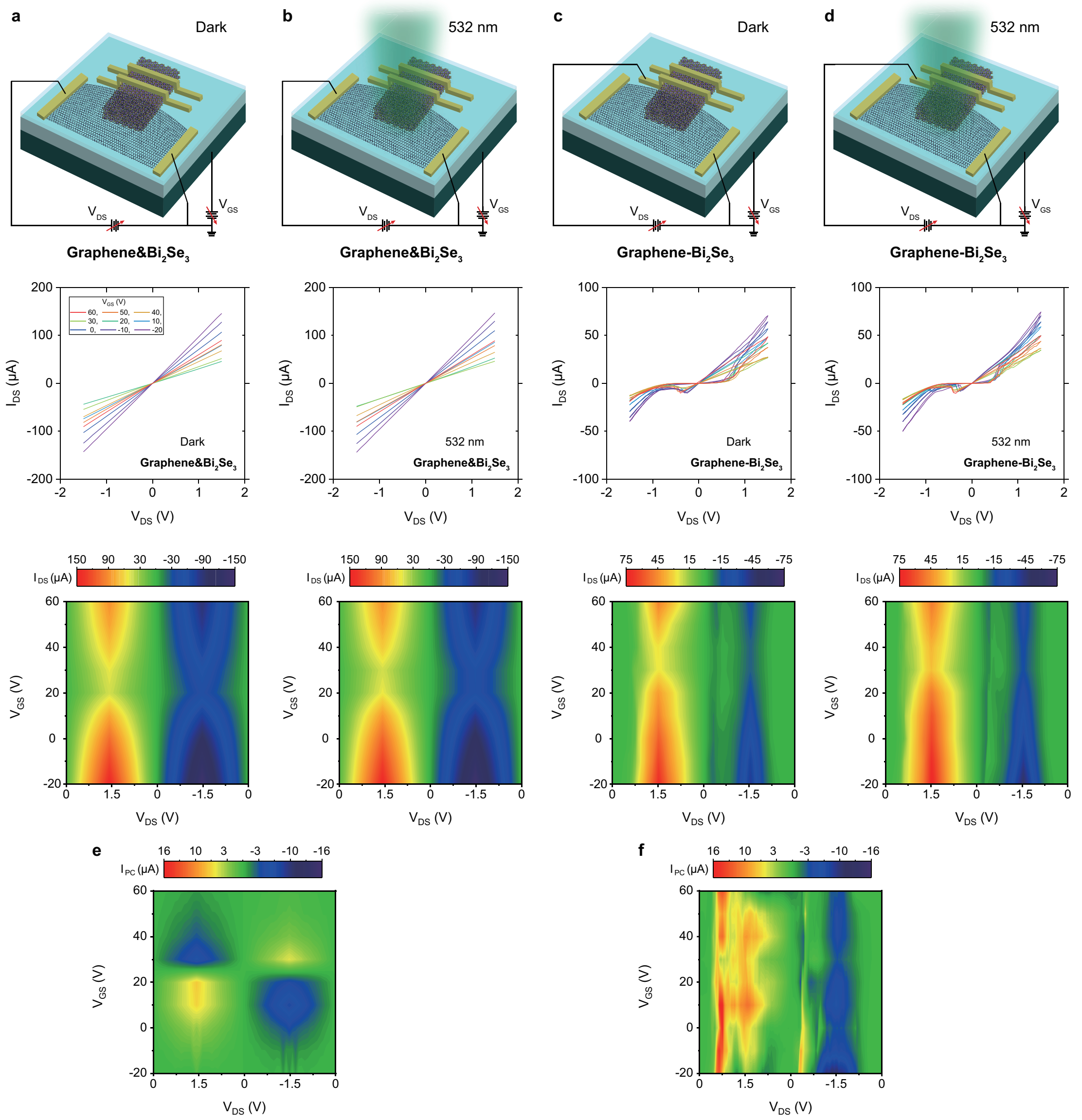

Figure S3: Photocurrent dependence on measurement configuration. **a-d**, Top: schematics of the different electrical connections on graphene or Bi_2Se_3 in dark (**a,c**) or under 532 nm light (power = 10 μW) illuminated onto the heterojunction (**b,d**). Middle: I_{DS}-V_{DS} curves of graphene&Bi_2Se_3 (**a,b**) and graphene-Bi_2Se_3 (**c,d**) at different V_{GS} with and without 532 nm laser illumination. **e,f**, Color plots of I_{PC} depending on V_{DS} and V_{GS} for graphene&Bi_2Se_3 (**e**) and graphene-Bi_2Se_3 (**f**).

FN tunneling plot analysis

The energy band alignments before equilibrium and between each step is drawn in **Figure 2**c based on the estimation of the dominant transport mechanism in Fig. **3** by fitting **Figure 2**a to the direct $\left(\textbf{Equation 1}, ln\left(\frac{I_{\mathrm{DT}}}{V_{\mathrm{DS}}{}^2}\right) = \frac{Aq^2\sqrt{m\Phi_{\mathrm{B}}}}{h^2 d} ln\left(\frac{1}{V_{\mathrm{DS}}}\right) - \frac{4\pi d\sqrt{2m^*\Phi_{\mathrm{B}}}}{h}\right)$ or FN $\left(\textbf{Equation 2}, ln\left(\frac{I_{\mathrm{FNT}}}{V_{\mathrm{DS}}{}^2}\right) = -\frac{8\pi d\sqrt{2m^*\Phi_{\mathrm{B}}{}^3}}{3hq}\left(\frac{1}{V_{\mathrm{DS}}}\right) + ln\left(\frac{Aq^3 m}{8\pi h\Phi_{\mathrm{B}} d^2 m^*}\right)\right)$ tunneling equations,[4–8] where I_{DT}, I_{FNT}, A, q, m, m^*, h, Φ_{B}, and d are the direct tunneling current, FN tunneling current, effective junction area, electric charge, free electron mass, effective electron mass, Planck constant, barrier height, and barrier width, respectively. According to **Equations 1** and **2**, the dominant transport mechanisms are determined in **Figure 3**a-h by analyzing the trends of each FN plot.[4–8] The total current is dominated by the direct tunneling, when the slope of $\ln(I_{\mathrm{DS}}/V_{\mathrm{DS}}{}^2)$ versus $1/V_{\mathrm{DS}}$ plot is positive and increases exponentially (**Equation 1**), which corresponds to **Figure 3**a,h. If $\ln(I_{\mathrm{DS}}/V_{\mathrm{DS}}{}^2)$ versus $1/V_{\mathrm{DS}}$ plot exhibits the negative and linear slope (**Equation 2**), the FN tunneling is dominant, as shown in **Figure 3**b,f,g. We extracted the effective tunneling barrier heights Φ_{B} on the graphene side ($\simeq$ 0.358 eV, **Figure 3**b,i) and Bi_2Se_3 side ($\simeq$ 0.660 eV, **Figure 3**f,j) from the slopes in the linear regions of each plot with **Equation 2**, providing direct evidence for the asymmetric tunneling barrier heights. The slope of $\ln(I_{\mathrm{DT}}/V_{\mathrm{DS}}{}^2)$ versus $\ln(|1/V_{\mathrm{DS}}|)$ plot (**Equation 1**) depends on the barrier height, which also varies with V_{DS}. Therefore, if $\ln(I_{\mathrm{DS}}/V_{\mathrm{DS}}{}^2)$ versus $\ln(|1/V_{\mathrm{DS}}|)$ plot reveals a linear increase rather than a logarithmic growth, it can be predicted that other charge carrier transport mechanisms are coupled with the tunneling process (**Figure 3**d,e). A transition from direct tunneling to FN tunneling is known to be realized only when the tunneling barrier is low in height and narrow in width,[4–8] which is observed at II and VII in our work.

Charge carrier trapping processes at the interface

The origin of hysteretic I-V characteristics shown in **Figure 2** is related to the charge trapping/detrapping processes at the graphene/Bi_2Se_3 interface. At the forward(reverse) bias voltage, the reverse(forward) sweep current is higher than the forward(reverse) sweep current, and the direction is anticlockwise(clockwise). At the low V_{DS} regime (I $\rightarrow$ II), the tunneling barrier is not strongly deformed by the external electric field so that the dominant mechanism will be the direct tunneling. When V_{DS} reaches to 0.45 V (II) transforming the barrier shape from trapezoid to triangle, the tunneling process will be dominated by FN tunneling across the triangular barrier (II $\rightarrow$ III). If the V_{DS} exceeds 0.78 V (III), the charge carriers will have enough energy to surmount the barrier height, and the thermionic emission will contribute to the total current (III $\rightarrow$ IV). The hole carriers trapped on the surface of the oxidation layer until $V_{DS} = 1.5$ V (IV), start to release during the reverse sweep (IV $\rightarrow$ V), and the reverse sweep current is increased accordingly. In consideration of the higher forward sweep current at the reverse bias voltage, the trapped holes are expected to remain until $V_{DS} = -1.12$ V (VII). The hump-like I-V curve in the region of V $\rightarrow$ VII is believed to occur because the direct tunneling current is suppressed due to the Fermi-level of Bi_2Se_3 crossing the Dirac point of graphene, where the density of states is zero. As the Fermi-level of Bi_2Se_3 shifts across the Dirac point of graphene, the negative differential resistance (NDR) effect[9,10] is observed (VI $\rightarrow$ VII), which is associated with the competition between the suppressed tunneling of electrons into the Dirac point of graphene and the tunneling of trapped holes into Bi_2Se_3. If the reverse bias voltage increases more (VII $\rightarrow$ VIII), the FN tunneling is found to be dominant. Unlike at the forward bias voltage (III $\rightarrow$ IV), the thermionic emission is not observed at the reverse bias voltage (until VIII), due to the higher barrier height for electrons on the Bi_2Se_3 side than that on the graphene side. Because of the narrow transient bias voltage region until $V_{DS} = -1.5$ V (VIII), the trapped electrons during the forward sweep are not enough to change the reverse sweep current (VIII $\rightarrow$ IX),

and the dominant mechanism will remain as the FN tunneling. When V_{DS} is swept back from IX to I, the direct tunneling will dominate again, and the band alignment returns to the original state.

Energy band alignment across the graphene/Bi_2Se_3 heterointerface

There are several things to note about the energy band diagram of the graphene/Bi_2Se_3 heterojunction. As shown in **Figure 2**c, the graphene grown by chemical vapor deposition is slightly p-doped due to the inevitable doping effects of substrates, defects, water molecules, and other chemical impurities introduced during the transfer process,[11–14] as confirmed by the positive Dirac voltage V_{Dirac} observed in the transfer curves (**Figure S4**). Based on the dispersion relation of graphene, the average Fermi-level shift from Dirac point of graphene is obtained as $\Delta E_{\mathrm{F0}} \simeq 0.161$ eV in dark. The average carrier mobilities of the graphene channel are obtained as $\simeq 562$ cm^{2}V^{-1}s^{-1} for holes and $\simeq 354$ cm^{2}V^{-1}s^{-1} for electrons at room-temperature. On the contrary, the Fermi-level of Bi_2Se_3 is known to be strongly pinned above the bulk conduction band edge due to Se vacancies on the surface.[15–17] The maximum field-effect mobility of the pure Bi_2Se_3 channel is obtained as $\simeq 14.7$ cm^{2}V^{-1}s^{-1} for electrons at room-temperature (**Figure S5**). Accordingly, under the gate bias voltage, the Fermi-level shifts will occur mostly in the graphene part, while the Fermi-level of Bi_2Se_3 remains almost unchanged. Note that the oxidation layer is analyzed only with Bi_2O_3 in **Figure 2**c to avoid confusion. Because of the Se vacancies on the Bi_2Se_3 surface,[15–17] the dominant oxidation layer formed at the graphene/Bi_2Se_3 interface will be Bi_2O_3 rather than SeO_2. This corresponds to the XPS results obtained in **Figure 1**f. Considering the electron affinity of SeO_2 (≈ 1.82 eV),[18] which is much lower than that of Bi_2O_3 (≈ 4.94 eV),[19] the heights and shapes of Bi_2O_3 tunneling barrier[19–21] are suitable for describing the transition of carrier transport mechanisms observed in **Figure 2**a,b and the tunneling barrier heights estimated in **Figure 3**. In reality, both Bi_2O_3 and SeO_2 will exist as tunneling barriers at the interface. In accordance with each energy level,[18–21] the transition of carrier transport mechanisms are caused by Bi_2O_3,[19] while SeO_2 contributes mostly to direct tunneling.[18] It is also expected that electrons will be injected from Bi_2Se_3 into graphene during the junction formation, and the band bending arises from graphene to Bi_2Se_3 at an equilibrium state,

bearing the asymmetric tunneling barrier heights. Owing to the conductive Dirac surface states of both graphene and Bi_2Se_3, the charge trapping will occur only at the interface between the van der Waals gap and oxidation layer of Bi_2Se_3. It should be also noted that the rectifying and hysteretic characteristics are found to degrade significantly after applying V_{DS} more than 2 V, which is attributed to the vanishing of the tunneling junction under a high electric field.

Photoresponse characteristics of the graphene&Bi_2Se_3

Figure S4a,b shows the transfer curves of the reference graphene transistor (graphene&Bi_2Se_3) in dark or under light illumination at various wavelengths of 532, 730, 1550, 4000 nm with a laser power of 10 μW at $V_{\mathrm{DS}} = 0.5$ V (**Figure S4**a) and $V_{\mathrm{DS}} = 1.5$ V (**Figure S4**b). The corresponding photoresponsivity and photodetectivity are shown in **Figure S4**c,d.

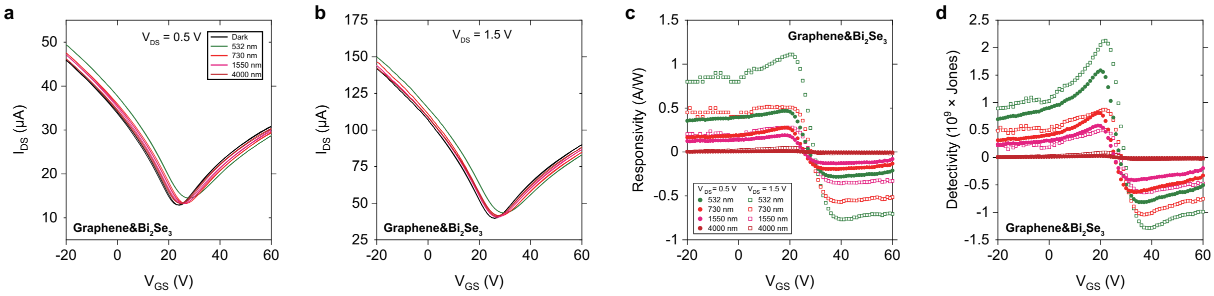

Figure S4: Photoresponse characteristics of the reference graphene transistor. **a,b**, I_{DS}-V_{GS} curves $V_{\mathrm{DS}} = 0.5$ (**a**) and $V_{\mathrm{DS}} = 1.5$ (**b**) in dark or under light illumination over a wide range of wavelengths (532 nm, 730 nm, 1550 nm, 4000 nm). **c,d**, Photoresponsivity (**c**) and detectivity (**d**) plots (**g,h**) as a function of V_{GS} at the different wavelengths.

Output and transfer curves of the Bi_2Se_3 field-effect transistor

Figure S5 shows the field-effect transistor characteristics of Bi_2Se_3 channel. From the transfer curve (**Figure S5**c), the maximum field-effect mobility is obtained as 14.7 $cm^2V^{-1}s^{-1}$ for electrons at room-temperature. Since it is unlikely for water or oxygen molecules to be adsorbed on the Bi_2Se_3 surface considering the Al_2O_3 passivation layer, the hysteresis effect in **Figure S5**c will be mainly attributed to the oxygen vacancies in the Bi_2Se_3 flake.[22]

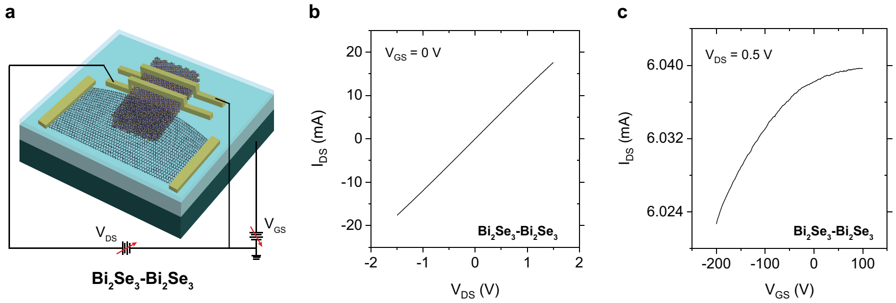

Figure S5: Output and transfer curves of the Bi_2Se_3 field-effect transistor. **a**, Schematics of the electrical connections on Bi_2Se_3-Bi_2Se_3 channel. **b**, I_{DS}-V_{DS} curve at V_{GS} = 0 V. **c**, I_{DS}-V_{GS} curve at V_{DS} = 0.5 V.

Photogating effect due to the trapping of photogenerated carriers

All the transfer curves (**Figure 4**e,f) shift the Dirac voltage V_{Dirac} towards the positive side in the graphene-Bi_2Se_3 heterochannel under light illumination. This is because, as shown in **Figure 4**b,c, a considerable number of the photoexcited holes generated in Bi_2Se_3 side can be trapped at the interface during the direct tunneling, while most of the photoexcited electrons generated in graphene side will be easily injected into the conduction band of Bi_2Se_3 over the low barrier. The trapped holes will act like additional V_{GS}, providing an electrostatic potential drop to the graphene layer.[23–26] This leads to the increased hole density in graphene and produces a horizontal shift of the Dirac point towards positive V_{GS}. From the shift of $\mathrm{V_{Dirac}}$, we estimate the photogating effect attributed to the trapping of photogenerated carriers at the graphene/Bi_2Se_3 interface. As shown in **Figure S6**a, the average Fermi-level shift from Dirac point of graphene (ΔE_{F0}: in dark and ΔE_{F}: under laser illumination) and the corresponding average carrier density of graphene (n_0: in dark and n: under laser illumination) are obtained at different laser wavelengths. The trap-assisted photoinduced carrier density of graphene can be defined as $\Delta n = n - n_0$. Higher photon energy results in higher Δn that leads to the conductance modulation in graphene channel. However, there was no significant change in Δn at between $V_{\mathrm{DS}} = 0.5$ and 1.5 V, indicating that the tunneling resistance does not show pronounced influence on photogating effect due to trapping of photogenerated carriers. This is because the tunneling resistance affects photovoltaic effect, while photogating effect originates in charge trapping of photoexcited holes at the interface. **Figure S6**b shows that the shift of $\mathrm{V_{Dirac}}$ mostly occurs at lower laser power (1 μW) and saturates with increasing laser power (10 μW), since the photogating effect is much significant at lower laser power due to the limited interface-trap density.[27] The operation at higher laser power is believed to be further improved by introducing the higher interface-trap density.

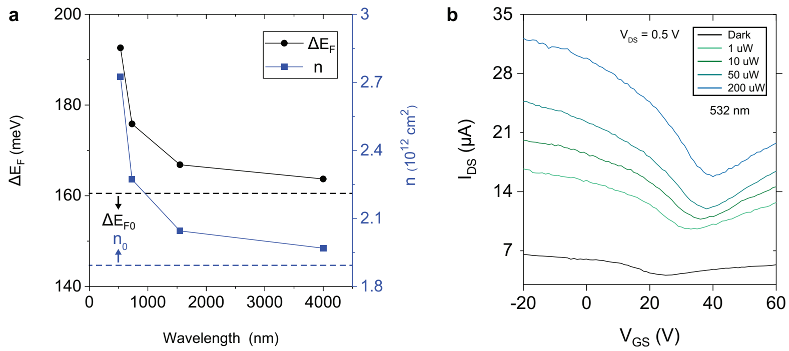

Figure S6: Wavelength- and power-dependent photogating effect. **a**, Dependence of Fermi-level shift and carrier density of the graphene-Bi_2Se_3 heterochannel on laser wavelength. ΔE_{F0} and ΔE_{F} are the average Fermi-level shifts from Dirac point in dark and under laser illumination. n_0 and n are the avergae carrier densities of graphene in dark and under laser illumination. **b**, Dependence of I_{DS}-V_{GS} curve of the graphene-Bi_2Se_3 heterochannel on laser power at $V_{\mathrm{DS}} = 0.5$ V.

Relation between light power and responsivity

Defined β as $R \simeq I^{\beta}$, the deviation of β from -1 represents the loss of photoexcited carriers, which increases with the light wavelength. This is because the injection rate of photoexcited carriers tunneling through the interfacial barrier or overcoming the barrier height decreases with the incident photon energy. The photoresponsivity decreases with incident light intensity for all wavelengths, which is known to be caused by the saturation of electron-hole pair generation and reduced recombination lifetime at higher light power.[27]

Photoswitch characteristics of the graphene-Bi_2Se_3

The rise I_{rise} and decay I_{decay} currents can be expressed as $I_{\text{rise}}(t) = I_{\text{dark}} + A\exp(t/\tau_{\text{rise}})$ and $I_{\text{decay}}(t) = I_{\text{dark}} + A\exp(-t/\tau_{\text{decay}})$, where I_{dark} is the dark current, A is the scaling constant, τ_{rise} is the rise time constant, τ_{decay} is the decay time constant, and t is the time when the light is switched on and off. The average values of τ_{rise} and τ_{decay} were estimated by fitting the rise and decay curves in **Figure S7**a and listed in **Figure S7**b. The response at the wavelength of 4000 nm is measured to be unstable so that the corresponding time constants were not able to be obtained. The faster photoresponse was achieved with shorter laser wavelengths.

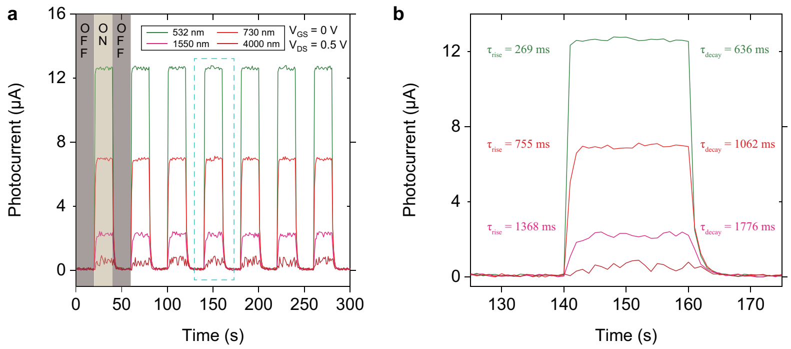

Figure S7: Time-resolved photoresponse characteristics of graphene-Bi_2Se_3. **a**, Time-resolved photocurrent in response to laser on and off at $V_{DS} = 0.5$ and $V_{GS} = 0$. Different colors denote the wavelengths of 532, 730, 1550, and 4000 nm. **b**, Zoom-in plot of the selected part indicated by the turquoise dashed square in **a**. The interval of switching time for laser on and off was set to 20 seconds.

References

(1) Steinberg, H.; Gardner, D. R.; Lee, Y. S.; Jarillo-Herrero, P., Surface State Transport and Ambipolar Electric Field Effect in Bi_2Se_3 Nanodevices. *Nano Lett.* **2010**, *10* (12), 5032-5036.

(2) Bansal, N.; Kim, Y. S.; Brahlek, M.; Edrey, E.; Oh, S., Thickness-Independent Transport Channels in Topological Insulator Bi_2Se_3 Thin Films. *Phys. Rev. Lett.* **2012**, *109* (11), 116804.

(3) Hasan, M. Z.; Kane, C. L., Colloquium: Topological Insulators. *Rev. Mod. Phys.* **2010**, *82* (4), 3045.

(4) Beebe, J. M.; Kim, B.; Gadzuk, J. W.; Frisbie, C. D.; Kushmerick, J. G., Transition from Direct Tunneling to Field Emission in Metal-Molecule-Metal Junctions. *Phys. Rev. Lett.* **2006**, *97* (2), 026801.

(5) Lee, G.-H.; Yu, Y.-J.; Lee, C.; Dean, C.; Shepard, K. L.; Kim, P.; Hone, J., Electron Tunneling through Atomically Flat and Ultrathin Hexagonal Boron Nitride. *Appl. Phys. Lett.* **2011**, *99* (24), 243114.

(6) Li, H.-M.; Lee, D.; Qu, D.; Liu, X.; Ryu, J.; Seabaugh, A.; Yoo, W. J., Ultimate Thin Vertical p–n Junction Composed of Two-Dimensional Layered Molybdenum Disulfide. *Nat. Commun.* **2015**, *6* (1), 1-9.

(7) Ahmed, F.; Choi, M. S.; Liu, X.; Yoo, W. J., Carrier Transport at the Metal–MoS_2 Interface. *Nanoscale* **2015**, *7* (20), 9222-9228.

(8) Vu, Q. A.; Lee, J. H.; Nguyen, V. L.; Shin, Y. S.; Lim, S. C.; Lee, K.; Heo, J.; Park, S.; Kim, K.; Lee, Y. H., Tuning Carrier Tunneling in van der Waals Heterostructures for Ultrahigh Detectivity. *Nano Lett.* **2017**, *17* (1), 453-459.

(9) Ren, H.; Li, Q.-X.; Luo, Y.; Yang, J., Graphene Nanoribbon as a Negative Differential Resistance Device. *Appl. Phys. Lett.* **2009**, *94* (17), 173110.

(10) Wu, Y.; Farmer, D. B.; Zhu, W.; Han, S.-J.; Dimitrakopoulos, C. D.; Bol, A. A.; Avouris, P.; Lin, Y.-M., Three-Terminal Graphene Negative Differential Resistance Devices. *ACS Nano* **2012**, *6* (3), 2610-2616.

(11) Pirkle, A.; Chan, J.; Venugopal, A.; Hinojos, D.; Magnuson, C.; McDonnell, S.; Colombo, L.; Vogel, E.; Ruoff, R.; Wallace, R., The Effect of Chemical Residues on the Physical and Electrical Properties of Chemical Vapor Deposited Graphene Transferred to SiO_2. *Appl. Phys. Lett.* **2011**, *99* (12), 122108.

(12) Yoon, H. H.; Jung, S.; Choi, G.; Kim, J.; Jeon, Y.; Kim, Y. S.; Jeong, H. Y.; Kim, K.; Kwon, S.-Y.; Park, K., Strong Fermi-Level Pinning at Metal/n-Si (001) Interface Ensured by Forming an Intact Schottky Contact with a Graphene Insertion Layer. *Nano Lett.* **2017**, *17* (1), 44-49.

(13) Jung, S.; Yoon, H. H.; Jin, H.; Mo, K.; Choi, G.; Lee, J.; Park, H.; Park, K., Reduction of Water-Molecule-Induced Current-Voltage Hysteresis in Graphene Field Effect Transistor with Semi-Dry Transfer Using Flexible Supporter. *J. Appl. Phys.* **2019**, *125* (18), 184302.

(14) Yoon, H. H.; Song, W.; Jung, S.; Kim, J.; Mo, K.; Choi, G.; Jeong, H. Y.; Lee, J. H.; Park, K., Negative Fermi-Level Pinning Effect of Metal/n-GaAs (001) Junction Induced by a Graphene Interlayer. *ACS Appl. Mater. Interfaces* **2019**, *11* (50), 47182-47189.

(15) Suh, J.; Fu, D.; Liu, X.; Furdyna, J. K.; Yu, K. M.; Walukiewicz, W.; Wu, J., Fermi-Level Stabilization in the Topological Insulators Bi_2Se_3 and Bi_2Te_3: Origin of the Surface Electron Gas. *Phys. Rev. B* **2014**,*89* (11), 115307.

(16) Spataru, C. D.; Léonard, F., Fermi-Level Pinning, Charge Transfer, and Relaxation of

Spin-Momentum Locking at Metal Contacts to Topological Insulators. *Phys. Rev. B* **2014**, *90* (8), 085115.

(17) Yan, B.; Zhang, D.; Felser, C., Topological Surface States of Bi_2Se_3 Coexisting with Se Vacancies. *Phys. Status Solidi RRL* **2013**, *7* (1-2), 148-150.

(18) Snodgrass, J.; Coe, J.; McHugh, K.; Freidhoff, C.; Bowen, K., Photoelectron Spectroscopy of Selenium-and Tellurium-Containing Negative Ions: SeO_2-, Se_2-, and Te_2. *J. Phys. Chem.* **1989**, *93* (4), 1249-1254.

(19) Fan, H.; Li, H.; Liu, B.; Lu, Y.; Xie, T.; Wang, D., Photoinduced Charge Transfer Properties and Photocatalytic Activity in $Bi_2O_3/BaTiO_3$ Composite Photocatalyst. *CS Appl. Mater. Interfaces* **2012**, *4* (9), 4853-4857.

(20) Ho, C.-H.; Chan, C.-H.; Huang, Y.-S.; Tien, L.-C.; Chao, L.-C., The Study of Optical Band Edge Property of Bismuth Oxide Nanowires α-Bi_2O_3. *Opt. Express* **2013**, *21* (10), 11965-11972.

(21) Takane, D.; Souma, S.; Sato, T.; Takahashi, T.; Segawa, K.; Ando, Y., Work Function of Bulk-Insulating Topological Insulator $Bi_{2˘x}Sb_xTe_{3-y}Se_y$. *Appl. Phys. Lett.* **2016**, *109* (9), 091601.

(22) Late, D. J.; Liu, B.; Matte, H. R.; Dravid, V. P.; Rao, C., Hysteresis in Single-Layer MoS_2 Field Effect Transistors. *ACS Nano* **2012**, *6* (6), 5635-5641.

(23) Konstantatos, G.; Badioli, M.; Gaudreau, L.; Osmond, J.; Bernechea, M.; De Arquer, F. P. G.; Gatti, F.; Koppens, F. H., Hybrid Graphene–Quantum Dot Phototransistors with Ultrahigh Gain. *Nat. Nanotechnol.* **2012**, *7* (6), 363-368.

(24) Liu, Y.; Wang, F.; Wang, X.; Wang, X.; Flahaut, E.; Liu, X.; Li, Y.; Wang, X.; Xu, Y.; Shi, Y., Planar Carbon Nanotube–Graphene Hybrid Films for High-Performance Broadband Photodetectors. *Nat. Commun.* **2015**, *6* (1), 1-7.

(25) Fang, H.; Hu, W., Photogating in Low Dimensional Photodetectors. *Adv. Sci.* **2017**, *4* (12), 1700323.

(26) Kim, H. J.; Lee, K. J.; Park, J.; Shin, G. H.; Park, H.; Yu, K.; Choi, S.-Y., Photoconductivity Switching in $MoTe_2$/Graphene Heterostructure by Trap-Assisted Photogating. *ACS Appl. Mater. Interfaces* **2020**, *12* (34), 38563-38569.

(27) Yu, W. J.; Liu, Y.; Zhou, H.; Yin, A.; Li, Z.; Huang, Y.; Duan, X., Highly Efficient Gate-Tunable Photocurrent Generation in Vertical Heterostructures of Layered Materials. *Nat. Nanotechnol.* **2013**, *8* (12), 952-958.
